# Mice With Decreased Number of Interneurons Exhibit Aberrant Spontaneous and Oscillatory Activity in the Cortex

**DOI:** 10.3389/fncir.2018.00096

**Published:** 2018-10-31

**Authors:** Katerina Kalemaki, Xanthippi Konstantoudaki, Simona Tivodar, Kyriaki Sidiropoulou, Domna Karagogeos

**Affiliations:** ^1^School of Medicine, University of Crete, Voutes University Campus, Heraklion, Greece; ^2^Institute of Molecular Biology and Biotechnology, Foundation for Research and Technology - Hellas, Heraklion, Greece; ^3^Department of Biology, University of Crete, Voutes University Campus, Heraklion, Greece

**Keywords:** MGE-derived interneurons, Rac1, synaptic plasticity, neuronal oscillations, high K^+^ aCSF

## Abstract

GABAergic (γ-aminobutyric acid) neurons are inhibitory neurons and protect neural tissue from excessive excitation. Cortical GABAergic neurons play a pivotal role for the generation of synchronized cortical network oscillations. Imbalance between excitatory and inhibitory mechanisms underlies many neuropsychiatric disorders and is correlated with abnormalities in oscillatory activity, especially in the gamma frequency range (30–80 Hz). We investigated the functional changes in cortical network activity in response to developmentally reduced inhibition in the adult mouse barrel cortex (BC). We used a mouse model that displays ∼50% fewer cortical interneurons due to the loss of Rac1 protein from Nkx2.1/Cre-expressing cells [Rac1 conditional knockout (cKO) mice], to examine how this developmental loss of cortical interneurons may affect basal synaptic transmission, synaptic plasticity, spontaneous activity, and neuronal oscillations in the adult BC. The decrease in the number of interneurons increased basal synaptic transmission, as examined by recording field excitatory postsynaptic potentials (fEPSPs) from layer II networks in the Rac1 cKO mouse cortex, decreased long-term potentiation (LTP) in response to tetanic stimulation but did not alter the pair-pulse ratio (PPR). Furthermore, under spontaneous recording conditions, Rac1 cKO brain slices exhibit enhanced sensitivity and susceptibility to emergent spontaneous activity. We also find that this developmental decrease in the number of cortical interneurons results in local neuronal networks with alterations in neuronal oscillations, exhibiting decreased power in low frequencies (delta, theta, alpha) and gamma frequency range (30–80 Hz) with an extra aberrant peak in high gamma frequency range (80–150 Hz). Therefore, our data show that disruption in GABAergic inhibition alters synaptic properties and plasticity, while it additionally disrupts the cortical neuronal synchronization in the adult BC.

## Introduction

The cerebral cortex consists of two main neuronal types, excitatory (pyramidal neurons, responsible for glutamate release) and inhibitory neurons (GABAergic interneurons, responsible for GABA release), which collaborate to organize, regulate and synchronize the flow of information through neuronal networks. The formation of functional networks through synchronized oscillation frequencies critically depends upon excitation to inhibition balance (E/I balance) (Haider and McCormick, 2009; Uhlhaas and Singer, 2013). Abnormalities in neuronal oscillations, particularly those in the range of gamma frequencies (30–80 Hz), are associated with many neuropsychiatric disorders (Herrmann and Demiralp, 2005). In particular, many studies on schizophrenia (SCZ) focus on gamma-frequency oscillations because of their significant contribution in cognitive functions (Tiitinen et al., 1993; Buzsáki and Draguhn, 2004). Fast synaptic inhibition mediated by GABA-A receptors underlies network synchrony and SCZ is associated with alterations in cortical GABAergic neurotransmission (Lewis et al., 2005; Sohal et al., 2009). GABAergic interneurons, especially the subpopulation expressing the calcium binding protein parvalbumin (PV), play a fundamental role in the generation and synchronization of gamma rhythms because of their fast-spiking characteristics and short time constants of synaptic interactions (Bartos et al., 2007; Sohal et al., 2009).

Numerous neuropsychiatric disorders, including epilepsy, depression, autism spectrum disorders (ASD) and SCZ exhibit E/I imbalance in the cortex (Lewis et al., 2003; Yizhar et al., 2011; Marin, 2012; Nelson and Valakh, 2015). Especially, a reduction in interneuron markers, such as GAD65/67 and PV have been correlated with several neuropsychiatric and neurological disorders, such as SCZ, ASD, depression and epilepsy as mentioned above (Sussel et al., 1999; Lewis et al., 2003, 2005, 2012; Markram et al., 2004; Kalueff and Nutt, 2007; Fatemi et al., 2008a,b; Lodge et al., 2009; Blatt and Fatemi, 2011; Hyde et al., 2011; Yizhar et al., 2011; Möhler, 2012; Powell, 2013). However, is it still unknown whether their alterations represent the cause of these pathologies or an adaptation of another, primary defect. Several transgenic mice which exhibit interneuron deficiencies have been used to demonstrate molecular components of interneuron development (Cobos et al., 2005; Butt et al., 2008; Kerjan et al., 2009; Close et al., 2012; Neves et al., 2012; Vidaki et al., 2012). These models could be used to resolve whether developmental deficiencies in the function of interneurons could underlie disease phenotypes.

In this report, we aim to investigate changes in local cortical network activities that occur in response to developmentally reduced inhibition. To this end, we use a transgenic mouse line generated in our group, in which the Rac1 gene is deleted from Nkx2.1-expressing neurons [Rac1^fl/fl^;Nkx2.1^Tg(Cre)^], from now on referred to as the Rac1 cKO mouse. These mice exhibit a 50% reduction in GABAergic interneurons expressing PV and somatostatin (SST) in the postnatal cortex, a decrease that results from a failure of interneuron progenitors originating in the medial ganglionic eminence (MGE) to exit the cell cycle (Vidaki et al., 2012). We focus on how this interneuron deficiency affects the synaptic properties and the local cortical neuronal synchronization. Our findings reveal that the adult cortex of Rac1 cKO mice has: (a) a severely decreased number of MGE-derived interneurons, (b) increased synaptic transmission and stimulus-evoked recurrent activity, (c) decreased long-term potentiation (LTP) but unaffected short-term potentiation, (d) enhanced spontaneous activity, (e) significant decrease in oscillatory activity of low frequency range, and (f) significant reduction and disorganization of gamma frequency range (30–80 Hz) with an aberrant peak at high gamma frequency range (80–150 Hz).

## Materials and Methods

### Animals and Housing

Adult male mice, 30–60 days of age, were used for all experiments. Mice were housed in groups (three to four per cage) and provided with standard mouse chow and water *ad libitum*, under a 12 h light/dark cycle (light on at 7:00 am) with controlled temperature (21 ±°C). The following genotypes were used for analysis: Rac1^fl/fl^;Nkx2.1^Tg(Cre)^;R26R-YFP^+/-^ (referred to as Rac1 cKO mice) and Rac1^+/fl^;Nkx2.1^Tg(Cre)^;R26R-YFP^+/-^ (referred as heterozygous mice). The heterozygous mice are used as the control group to Rac1 cKO as they present no differences when compared to wild type mice (Vidaki et al., 2012). The Rac1^fl/fl^;Nkx2.1^Tg(Cre)^ line has been previously described (Vidaki et al., 2012). Specifically, animals were generated carrying a floxed allele of Rac1 (Rac1^fl/fl^), where the fourth and fifth exon of the Rac1 gene has been flanked with loxP sites (Walmsley et al., 2003). These mice were crossed with Nkx2.1^Tg(Cre)^ mice [Nkx2.1-Cre transgenic (Fogarty et al., 2007)], so that Rac1^fl/fl^;Nkx2.1^Tg(Cre)^ mice were produced. The ROSA26fl-STOP-fl-YFP allele was also inserted to allow visualization via yellow fluorescent protein (YFP) expression (Srinivas et al., 2001) of the MGE-derived interneurons where Rac1 is deleted. Mice used in these experiments resulted from crossing Rac1^fl/fl^;Nkx2.1^+/+^;R26R-YFP^+/-^ with Rac1^+/fl^;Nkx2.1^Tg(Cre)^;R26R-YFP^+/-^ genotypes. At least 80% of heterozygous and Rac1 cKO animals originated from the same litters. More than half of the Rac1^fl/fl^;Nkx2.1^Tg(Cre)^;R26R-YFP^+/-^ (Rac1 cKO) die within 3 weeks after birth (Konstantoudaki et al., 2016). We performed experiments with mice that survived until postnatal day 60 (PD 60). All procedures were performed according to the European Union ethical standards and the IMBB and University of Crete ethical rules.

### Slice Preparation

Mice were decapitated under halothane anesthesia. The brain was removed promptly and placed in ice cold, oxygenated (95% O_2_–5% CO_2_) artificial cerebrospinal fluid (aCSF) containing (in mM): 125 NaCl, 3.5 KCl, 26 NaHCO_3_, 1 MgCl_2_ and 10 glucose (pH = 7.4, 315 mOsm/l). The brain was blocked and glued onto the stage of a vibratome (Leica, VT1000S). 400 μm thick coronal brain slices corresponding to distinct bregma along the rostrocaudal axis (–1.94 and 2) were selected, all including the BC region. The brain slices were taken and transferred to a submerged chamber, which was continuously superfused with oxygenated (95% O_2_–5% CO_2_) aCSF containing (in mM): 125 NaCl, 3.5 KCl, 26 NaHCO_3_, 2 CaCl_2_, 1 MgCl_2_ and 10 glucose (pH = 7.4, 315 mOsm/l) at room temperature (RT). The slices were allowed to equilibrate for at least 1 h in this chamber before recordings began. Slices were then transferred to a submerged recording chamber, continuously superfused oxygenated (95% O_2_–5% CO_2_) aCSF (same constitution as the one used for maintenance of brain slices) at RT during recordings.

### Electrophysiological Data Acquisition

All electrophysiological recordings were performed in both genotypes under the same conditions explained below. Extracellular recording electrodes filled with NaCl (2M) were placed in layers II/III of BC. Platinum/iridium metal microelectrodes (Harvard apparatus United Kingdom, 161 Cambridge, United Kingdom) were placed on layer II of the BC, about 300 μm away from the recording electrode, and were used to evoke field excitatory postsynaptic potentials (fEPSPs). Local field potentials (LFPs) were amplified using an extracellular headstage with selectable high pass filter of 30 Hz, to remove any offsets and a notch filter to eliminate line frequency noise, and gain of 100, coupled to a Dagan BVC-700A amplifier and low-pass filtered at 1-kHz. Signals were digitized using the ITC-18 board (InstruTech, Inc.) on a PC with custom-made procedures in IgorPro (Wavemetrics, Inc.) and stored on PC hard drive. All voltage signals were collected with a sampling frequency of 100 kHz (*F*_s_ = 100 kHz).

For evoked fEPSPs, the electrical stimulus consisted of a single square waveform of 100 μs duration given at intensities of 0.1–0.3 mA (current was increased from 0.1 mA to 0.3 mA, with 0.1 mA steps) generated by a stimulator equipped with a stimulus isolation unit (World Precision Instruments, Inc.). For paired-pulse recordings, two pulses were given at 10, 20, 50 Hz.

For the LTP experiments, baseline responses were acquired for 20 min (after 10 min of quiet period), then three 1-s tetanic stimuli (100 Hz) with an inter-stimulus interval of 20 s were applied, and finally responses were acquired for 50 min post-tetanus every 1-min.

For spontaneous recordings, 50 spontaneous voltage traces, of 5 s duration, were acquired under each of the following experimental condition: control aCSF and 0 Mg^++^ ions aCSF (Figure 4); control aCSF, high K^+^ aCSF and high K^+^ aCSF plus 2 μM diazepam (a GABA-A receptor agonist) in four heterozygous and four Rac1 cKO mice (Figure 5).

The control aCSF used in all electrophysiological experiments (evoked and spontaneous recordings) contained (in mM): 125 NaCl, 3.5 KCl, 26 NaHCO_3_, 2 CaCl_2_, 1 MgCl_2_ and 10 glucose (pH = 7.4, 315 mOsm/l). The 0 Mg^++^ aCSF used in specific spontaneous activity recordings contained (in mM): 125 NaCl, 3.5 KCl, 26 NaHCO_3_, 2 CaCl_2_, and 10 glucose (pH = 7.4, 315 mOsm/l), and the high K^+^ aCSF, also used in specific spontaneous activity recordings, contained (in mM): 125 NaCl, 7.5 KCl, 26 NaHCO_3_, 2 CaCl_2_ and 10 glucose (pH = 7.4, 315 mOsm/l) at RT. The contribution of GABA-A receptor activation was investigated by bath application of 2 μM Diazepam. Diazepam was acquired from the Pharmacy of the University General Hospital in Heraklion as a 5 mg/ml solution, and was diluted in high K^+^ aCSF during recordings.

### Electrophysiological Data Analysis

Data were analyzed using custom-written procedures in IgorPro software (Wavemetrics, Inc.). No additional high-pass filters were applied to the raw data.

For evoked recordings, the field peak values of the fEPSP were measured from the minimum value of the synaptic response (4–5 ms following stimulation) compared to the baseline value prior to stimulation. Both parameters were monitored in real-time in every experiment. A stimulus–response curve was then determined using stimulation intensities between 0.1 and 0.3 mA, in 0.1 mA steps. For each different intensity level, two traces were acquired and averaged. Baseline stimulation parameters were selected to evoke a response of 1 mV. To analyze the paired-pulse ratio, the fEPSP peak of the second pulse was divided to the fEPSP peak of the first pulse, for each different frequency (10, 20, and 50 Hz) of paired-pulse stimulation. For the LTP experiments, synaptic responses were normalized to the average 10 min pre-tetanic fEPSP. For the stimulus-induced recurrent discharge analysis, the first derivative of the voltage response was taken and the logarithm of its histogram was plotted (Dyhrfjeld-Johnsen et al., 2010).

In order to measure spontaneous activity events, the acquired spontaneous activity voltage signals of 5 s duration was decimated (down-sampled) by a factor of 10. To identify spontaneous events, the standard deviation σ_b_ of background signal was calculated in the ‘quiet’ part of each voltage response trace. To identify the ‘quiet’ period, each 5 s trace was split into 100 ms increments and the range of voltage deflection was computed in each increment. The ‘quiet’ part of the LFP trace was the 100 ms increment with the smallest σ_b_ value. As a spontaneous event, any voltage response larger than 4 σ_b_ was identified. We calculated the frequency of spontaneous events by measuring the number of spontaneous events divided by the duration of the trace (5 s). The frequency was calculated in 50 consecutive 5-s traces and then averaged for each animal. The spontaneous events do not correspond to spiking of individual neurons, they rather reflect population spikes.

### Detection of Oscillations

The time series for oscillations analysis were acquired using an extracellular headstage with selectable high pass filter of 30 Hz and gain of 100, to remove any offsets and a notch filter to eliminate line frequency noise, coupled to a Dagan BVC-700A amplifier and low-pass filtered at 1 kHz, as described above in the “Electrophysiological data acquisition” section.

For each genotype (i.e., heterozygous and Rac1 cKO mice) and for three experimental conditions [(1) control aCSF, (2) high K^+^ aCSF and (3) high K^+^ aCSF plus 2 μM diazepam] four replicates of 50 consecutive spontaneous voltage traces (each one of them voltage signals of 5 s duration, consequently total LFP trace of 250 s) with sampling rate 100 kHz acquired as described above. Each of the spontaneous voltage trace (time series) was transferred from time domain to frequency domain through Discrete Fourier Transformations and the power spectrum of the oscillation, for each frequency range, was computed. The ratios of the power spectrum from each frequency range were computed with respect to the power spectrum of all frequencies. The different frequency ranges for which the ratio of the power spectrum was computed were Delta: 1–4 Hz, Theta: 4–7 Hz, Alpha: 8–12 Hz, Beta: 13–30 Hz, Gamma: 30–80 Hz, High Gamma: 80–150 Hz and Total Gamma: 30–150 Hz. The use 30 Hz high-pass filter could have reduced the detection of the low frequency oscillations.

This measure is referred as the rate of power (%) and represents the percentage of the signal power spectrum (mV^2^/Hz) of each oscillation frequency range in relation to the power spectrum of whole signal (mV^2^/Hz). Therefore, the value of rate of power is without units and is computed by equation:

$$Rate\;of\;Power(\%)=sum\frac{Power\;(specific\;oscillation)}{Total\;(Power)}\%$$

This represents the signal rate of each oscillation in relation to the whole signal. All analysis was generated using custom-written procedures in MATLAB-R2015b (The MathWorks, Inc.), which can be found in the following link^1^.

### Immunohistochemistry

Adult mice (PD 30–60) were perfused with 4% PFA, followed by fixation with the same solution for 1 h at 4°C. They were subsequently processed as previously described (Vidaki et al., 2012). Primary antibodies used were rat monoclonal anti-GFP (Nacalai Tesque, Kyoto, Japan, 1:500) and rabbit polyclonal anti-PV (Swant, Bellinzona, Switzerland; 1:1000). Secondary antibodies used were goat anti-rat-Alexa Fluor-488 and goat anti-rabbit-Alexa Fluor-555 (Molecular Probes, Eugene, OR, United States, 1:800). For quantification of GFP and PV interneurons in adult mice, at least three pairs of littermate animals were used (heterozygous vs. Rac1 cKO). Images were obtained with a confocal microscope (Leica TCS SP2, Leica, Nussloch, Germany). For each pair, three sections corresponding to distinct bregma along the rostrocaudal axis (-1.94 and 2) were selected, all including the primary somatosensory barrel cortex (BC) field. PV and GFP positive cells in the BC were counted and an average rostrocaudal number was calculated for the interneuron subpopulations of heterozygous and Rac1 cKO animals.

### Nissl Staining

Brains of heterozygous and Rac1 cKO mice (PD 30–60) were removed, and placed in 4% PFA. After 24 h, the brains were place in PBS with 0.1% azide (at 4°C), until slicing. Brains were glued onto the vibratome stage and 40 μm-thick slices were acquired from three Rac1 heterozygous and three Rac1 cKO animals (VT1000S, Leica Microsystems, Wetzlar, 257 Germany). For each animal, three to four sections were used, corresponding to different rostrocaudal levels of the brain (-1.94 and 2), all including the primary somatosensory BC. Sections were incubated in xylene for 5 min and then for 3 min in 90%, 70% ethanol solutions and dH_2_O, followed by a 10-min incubation in 0,1% Cresyl violet solution. Sections were then dehydrated with increasing concentrations of ethanol (70%, 90%, 100%), incubated in xylene for 5 min and coverslipped with permount. Images from whole sections were obtained in 5× magnification of a light microscope (Axioskop 2FS, Carl Zeiss AG, 268 Oberkochen, Germany) and merged using Adobe Photoshop CC 2015, Adobe Systems, Inc.

### Statistical Analysis

#### Electrophysiology Recordings

One-way, two-way or repeated measures ANOVA or *t*-tests were performed depending on the experiment. One-way and two-way ANOVA test for spontaneous and oscillatory activity analysis performed by comparison between groups and within groups with Tukey *post hoc* test. Statistical analysis was performed in Microsoft Office Excel 2007, GraphPad Prism 6 or with IBM SPSS Statistics v.21. Data are presented as mean ± standard error of mean (SEM).

#### Cell Counting

The effect of the genotype on each subpopulation was assessed using ANOVA for repeated measurements and Student’s *t*-test. Data are presented as mean ± SEM.

## Results

### Decreased Numbers of Interneurons in the Adult Cortex of the Rac1 cKO Mouse

We studied the effect of embryonically initiated deficits of GABAergic interneurons on the adult BC using the Rac1 cKO mice. The Rac1 protein was eliminated from Nkx2.1-expressing cells using Cre/loxP recombination (Vidaki et al., 2012). In the nervous system, at embryonic day (E)9, Nkx2.1 starts to be expressed in MGE-derived cells (Sussel et al., 1999) and the elimination of Rac1 protein is obvious from MGE-derived cells by E12 (Vidaki et al., 2012). Consequently, the Rac1 protein is not expressed in MGE-derived interneurons, which are fated to become PV- and SST-positive interneurons in the Rac1 cKO BC (Vidaki et al., 2012).

**FIGURE 1:**
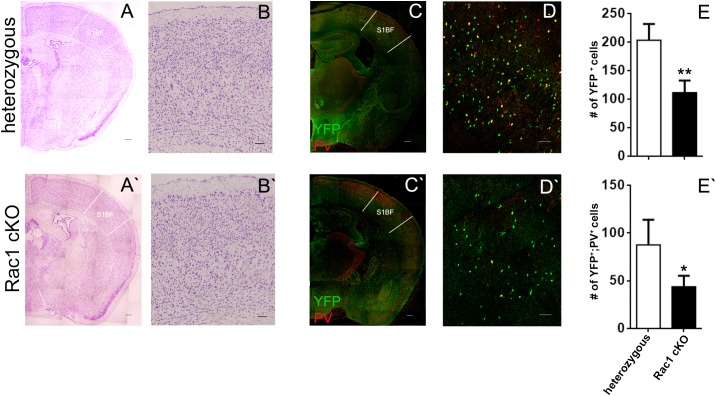
The number of MGE-derived cortical interneurons in the Rac1 cKO adult barrel cortex is severely reduced. **(A,A’)** Nissl staining of coronal sections of the adult cortex from heterozygous and Rac1 cKO. **(B,B’)** Representative areas of the barrel cortex from heterozygous and Rac1 cKO mice. **(C,C’)** Coronal sections from heterozygous and Rac1 cKO mice immunostained using anti-GFP and anti-PV antibodies. **(D,D’)** Representative areas of cell count from heterozygous and Rac1 cKO mice. **(E,E’)** Graphs showing that the number of YFP-positive and YFP;PV-double positive interneurons was reduced in the Rac1 cKO mice compared to heterozygous mice (*t*-test, *p* = 0.001, *n* = 6 slices from three heterozygous and *n* = 6 slices from three Rac1 cKO mice). Scale bars **(A,A’)** 200 μm; **(B,B’)** 100 μm; **(C,C’)** 300 μm; **(D,D′)** 75 μm.

Nissl staining on the BC slices from heterozygous and Rac1 cKO mice exhibited no gross anatomical defects (Figures 1A,A’,B,B’). Our previous studies showed that Rac1 cKO exhibit a significant reduction of MGE-derived interneurons in the juvenile (P15) somatosensory cortex and the adult prefrontal cortex (Vidaki et al., 2012; Konstantoudaki et al., 2016). Here, we investigated the number and distribution of interneurons in the adult BC, using equivalent cryosections for the heterozygous and Rac1 cKO mouse BC at different levels throughout the rostrocaudal axis in the two genotypes (Figures 1C,C’,D,D’). We counted the positive cells for YFP and PV that marks a major subpopulation of cortical interneurons deriving from the MGE. We observed that the number of Nkx2.1-derived YPF-positive interneurons (GFP^+^ cells, Figure 1E) and the number of YFP-PV double-positive interneurons are significantly reduced in the BC of Rac1 cKO compared to heterozygous mice (Figure 1E’).

### Alterations in Basal Synaptic Transmission and LTP in the Rac1 cKO Cortex

**FIGURE 2:**
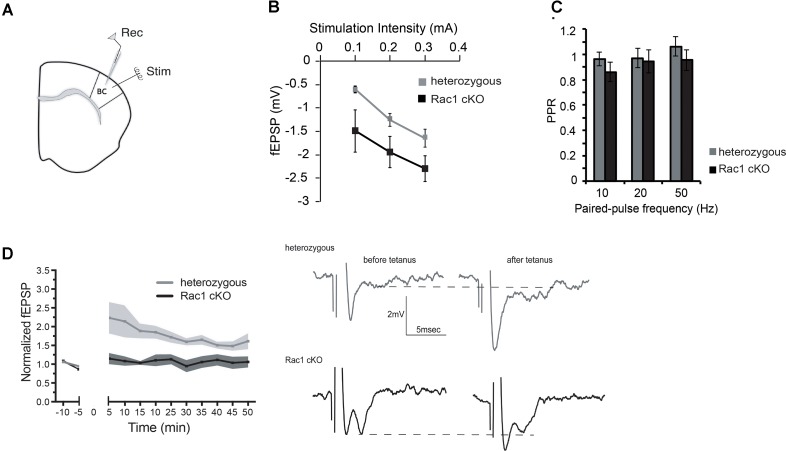
Increased basal synaptic transmission, unaltered paired-pulse ratio and decreased LTP in the Rac1 cKO cortex. **(A)** Schematic showing the position of the electrodes in BC brain slices (Rec: recording electrode, Stim: stimulating electrode). **(B)** Graph showing increased fEPSP responses of Rac1 cKO mice (*n* = 15 slices from seven Rac1 cKO mice), compared to heterozygous mice (*n* = 13 slices from eight heterozygous mice) [two-way repeated measures ANOVA, *F*(_1,14)_ = 0.5, *p* = 0.02]. **(C)** Graph showing no difference in the paired-pulse ratio when applying paired pulses of increasing frequency (10, 20, and 50 Hz) between heterozygous (*n* = 13 slices from eight heterozygous mice) and Rac1 cKO mice (*n* = 15 slices from seven Rac1 cKO mice) within layer II of barrel cortex [two-way repeated measures ANOVA, *F*_(1,14)_ = 3.94, *p* = 0.3]. **(D)** Graph (left) and representative traces (right) showing that in heterozygous mice (*n* = 5), tetanic stimulation results in enhanced fEPSP for at least 45 min, but in Rac1 cKO mice (*n* = 4), the enhanced response following tetanus is significantly smaller [two-way repeated measures ANOVA, *F*_(1,8)_ = 7.31, *p* = 0.01].

We next investigated whether the evoked synaptic properties of the Rac1 cKO cortex were altered. To study basal synaptic transmission, we delivered current pulses of increasing intensity through the stimulating electrode in BC layer II (Figure 2A) and recorded fEPSPs in BC brain slices. Rac1 cKO mice showed increased evoked fEPSP responses compared to heterozygous mice in the BC (Figure 2B), as expected in the presence of decreased number of interneurons. In order to further study the properties of synaptic responses, we delivered paired stimulations of different frequencies (10, 20, and 50 Hz). We found that the paired-pulse ratio (PPR) was not different between heterozygous and Rac1 cKO mice (Figure 2C). We then examined the ability of synapses to undergo LTP. In heterozygous brain slices, tetanic stimulation resulted in 50% increase of baseline fEPSP responses for at least 45 min. On the other hand, in Rac1 cKO BC slices, the same tetanic stimulation did not result in fEPSP potentiation (Figure 2D). Our results, so far, show that the neocortex of mice with decreased numbers of interneurons exhibit increased synaptic responses (fEPSPs), unaltered short-term synaptic plasticity and decreased LTP.

### Enhanced Activity in the Rac1 cKO Cortex

**FIGURE 3:**
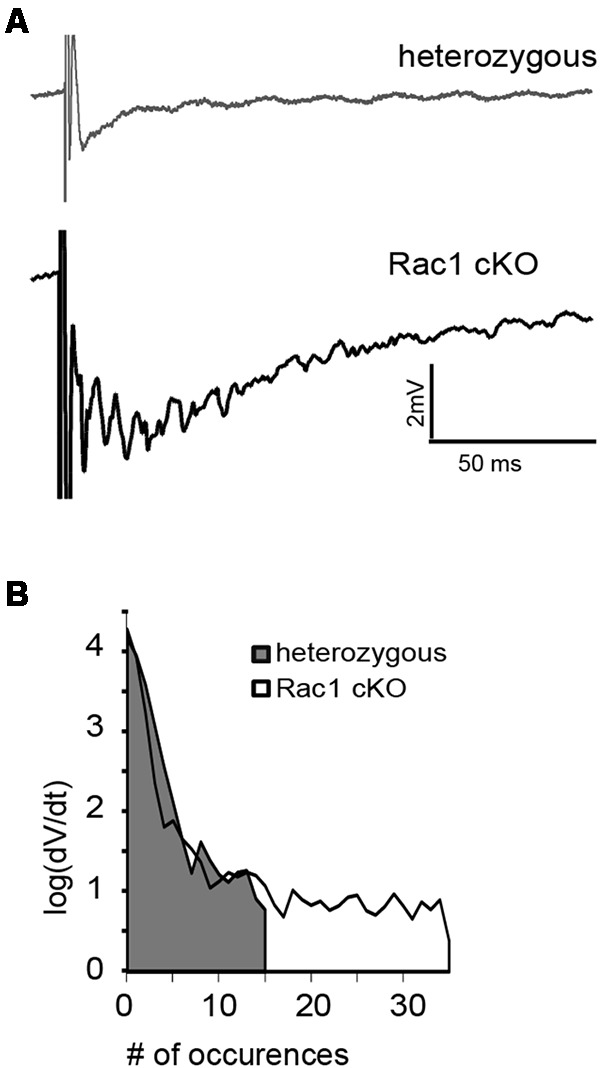
Identification of stimulus-induced recurrent activity in the Rac1 cKO cortex. **(A)** Stimulus-induced recurrent activity was evident when recording from Rac1 cKO neocortical slices, but not from heterozygous neocortical slices. **(B)** The histogram of the first derivative of the voltage response following stimulation extended to higher values in the stimulus-induced recurrent activity of Rac1 cKO slices, compared to that of heterozygous neocortical slices (*n* = 15 slices from seven Rac1 cKO mice; *n* = 13 slices from eight heterozygous mice).

**FIGURE 4:**
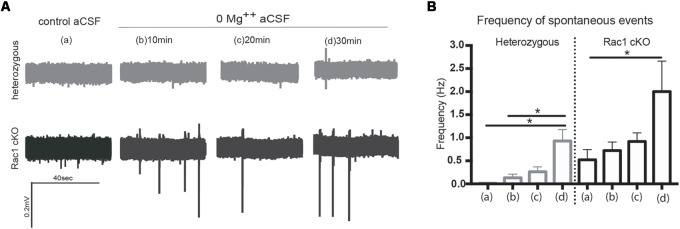
Rac1 cKO mice exhibit increased spontaneous activity. **(A)** Voltage traces from spontaneous activity recordings in heterozygous brain slices show that few spontaneous events (discharges) emerge under control aCSF or 0 Mg^++^ ions aCSF. In Rac1 cKO brain slices, voltage traces indicate increased frequency of spontaneous discharges when the Mg^++^ ions were removed from the aCSF solution. **(B)** Graph showing the number of spontaneous events per 5 s in control aCSF **(a)**, and 10 min **(b)**, 20 min **(c)**, and 30 min **(d)** following perfusion of 0 Mg^++^ ions aCSF solution from heterozygous and Rac1 cKO acute brain slices. The frequency of spontaneous events that emerged in brain slices from Rac1 cKO mice was significantly greater compared to the ones emerged in brain slices from heterozygous mice (*n* = 15 slices from seven Rac1 cKO mice; *n* = 13 slices from eight heterozygous mice) [one-way ANOVA, *F*_(1,28)_ = 2.104, *p* = 0.01, comparison between groups and within groups with Tukey *post hoc* test].

**FIGURE 5:**
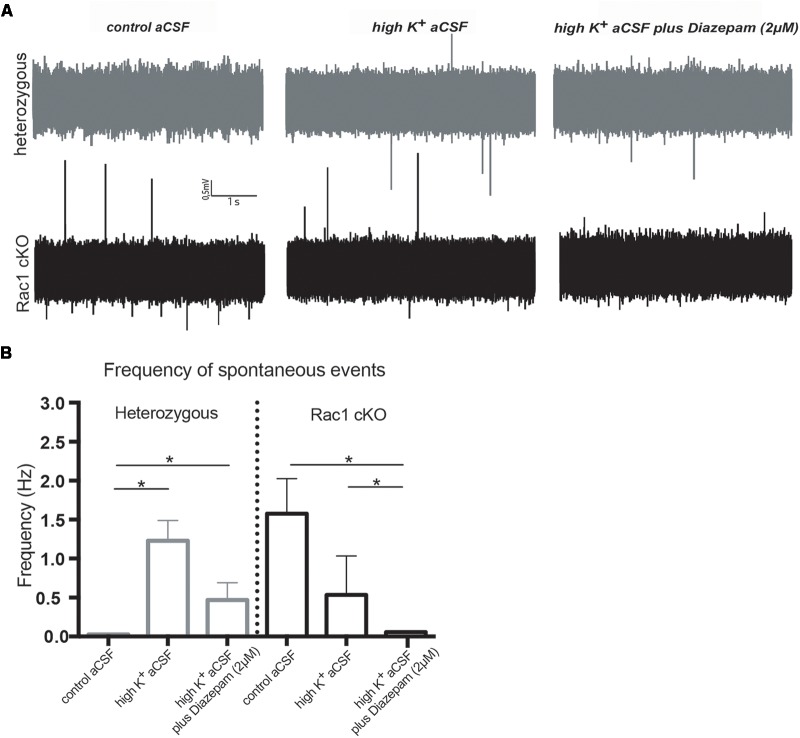
Rac1 cKO mice display increased susceptibility to induction of spontaneous events in the hyper-excitable brain slice. **(A)** Representative voltage traces from spontaneous activity recordings from heterozygous (top) and Rac1 cKO (bottom) brain slices in three conditions: (a) perfusion of control aCSF (left), (b) perfusion of high K^+^ aCSF for 20–30 min (middle) and (c) perfusion of high K^+^ aCSF plus 2 μM diazepam (a GABA-A receptor agonist) for 20–30 min (right). **(B)** Graph showing the frequency of spontaneous events in control aCSF, high K^+^ aCSF and in K^+^ aCSF plus diazepam conditions. The frequency of spontaneous events that emerged in Rac1 cKO brain slices was significantly greater compared to the ones emerged in heterozygous brain slices bathed control aCSF. The frequency of spontaneous events is statistically increased in high K^+^ aCSF, compared to control aCSF, and decreased in high K^+^ aCSF plus diazepam, compared to high K^+^ alone. In Rac1 cKO brain slices the frequency of spontaneous events remained unaltered in high K^+^ aCSF, compared to control aCSF, and significantly decreased in high K^+^ aCSF plus diazepam, compared to high K^+^ aCSF (*n* = 15 slices from seven Rac1 cKO mice; *n* = 13 slices from eight heterozygous mice) [one-way ANOVA, *F*_(1,15)_ = 1.625, *p* = 0.05, comparison between groups and within groups with Tukey *post hoc* test].

During our evoked fEPSP recordings, we observed recurrent discharges following the synaptic response in the Rac1 cKO brain slices, which was not evident in brain slices from heterozygous mice (Figure 3A). In order to graphically present these discharges in the two genotypes, we plotted the histogram of the first derivative of the voltage response following stimulation. This graphical representation indicates extended spiking activity in Rac1 cKO mice (Figure 3B). To better investigate this enhanced activity, we acquired spontaneous activity data from brain slices and measured spontaneously occurring events, in 50 continuous 5 s traces. We found significantly more spontaneous events in Rac1 cKO mice, compared to heterozygous, during control aCSF bath application [Figures 4A,B(a)]. We then used aCSF with 0 mM Mg^++^ ions, which renders the brain slice more excitable, and found that the number of spontaneous events that emerged in Rac1 cKO brain slices, was significantly and progressively greater compared to the heterozygous case [Figures 4A,B(b–d)]. Finally, we tested a third, even more excitable condition by increasing the extracellular concentration of potassium in aCSF (high K^+^ aCSF). We found that exposure to high K^+^ aCSF (for 20–30 min) significantly increased the number and frequency of spontaneous events in heterozygous, but not in Rac1 cKO brain slices (Figures 5A,B). It is possible that Rac1 cKO brain slices are more susceptible to spontaneous activity saturation and become rapidly unable to produce further spontaneous discharges. These results indicate enhanced spontaneous activity of the cortex with reduced numbers of interneurons, which reaches a plateau easier.

We further used diazepam, a GABA-A receptor agonist, during high K^+^ aCSF bath application, in order to increase inhibition in this excitable brain slice. We found a significant decrease in the number and frequency of spontaneous events both in Rac1 cKO and heterozygous genotypes (Figures 5A,B). These results suggest that both heterozygous and Rac1 cKO mice respond similarly to GABA-A receptor activation, during spontaneous activity.

### Different Oscillatory Activities Prevail in the Rac1 cKO

**FIGURE 6:**
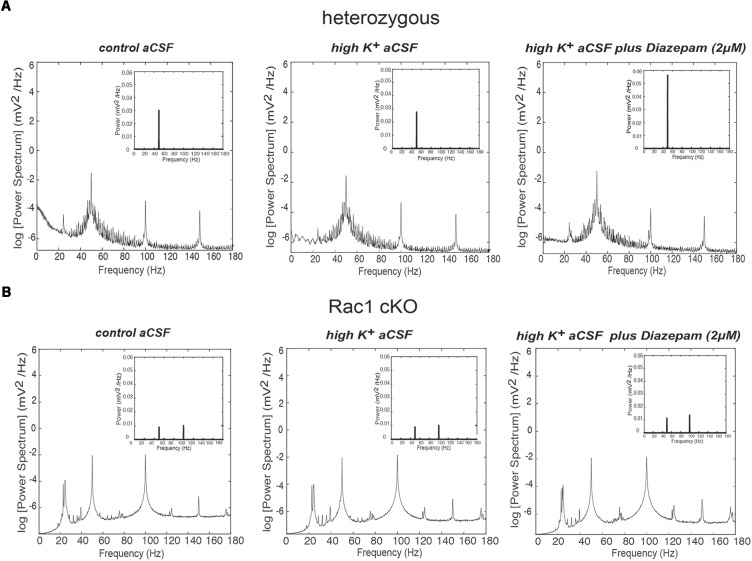
Power spectral density analysis in Rac1 cKO and heterozygous brain slices. **(A)** Power spectral density profiles in logarithmic scaling (bottom) and linear scaling (inset) in heterozygous brain slices, perfused with control aCSF (left), high K^+^ aCSF (middle) and high K^+^ aCSF plus diazepam (right) (*n* = 5 slices from five heterozygous mice). **(B)** Power spectral density profiles in logarithmic scaling (bottom) and linear scaling (inset) in Rac1 cKO brain slices perfused with control aCSF (left), high K^+^ aCSF (middle) and high K^+^ aCSF plus diazepam (right) (*n* = 5 slices from five Rac1 cKO mice).

Disrupted function of interneurons in a local neuronal network leads to altered synchronization across different frequency bands. Thus, we analyzed the power spectra of the recorded spontaneous activity (Figures 6A,B) in the three different conditions (control aCSF, high K^+^ aCSF and high K^+^ aCSF plus diazepam), as mentioned above. We observed differences in the shape and number of peaks observed between the Rac1 cKO and heterozygous mice. On the other hand, we did not observe significant variation among the three different conditions within either the heterozygous or the Rac1 cKO genotypes (Figures 6A,B).

**FIGURE 7:**
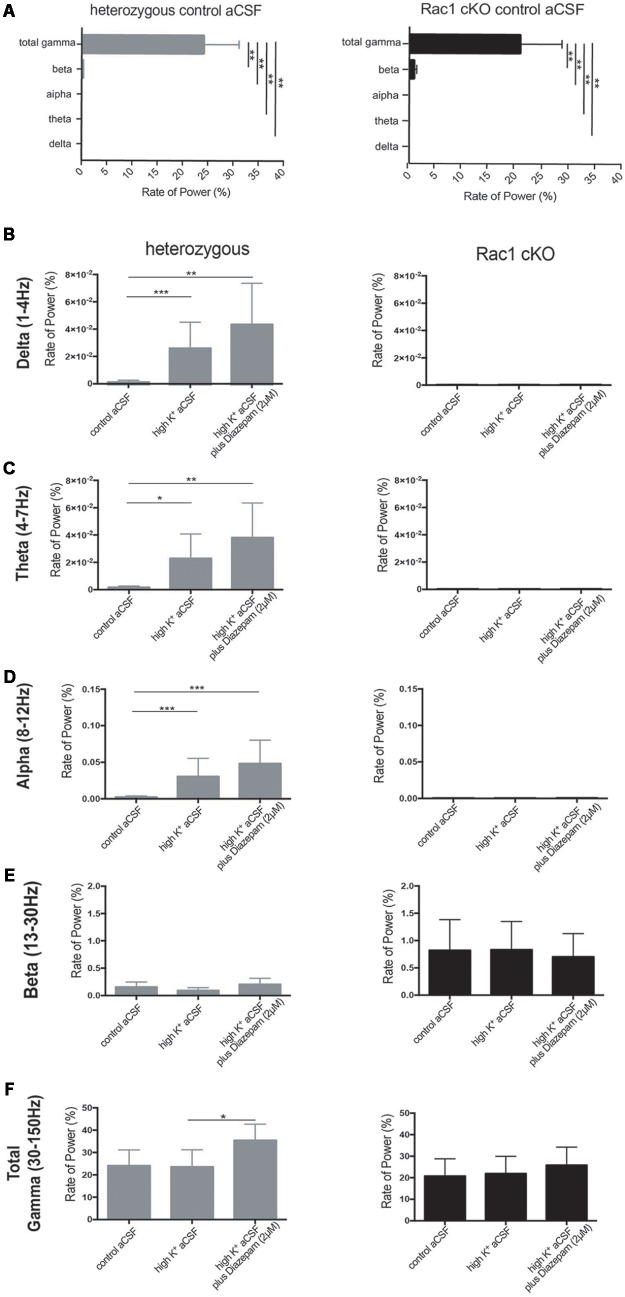
Rac1 cKO brain slices exhibited altered oscillatory activities. The rate of power (%) (see Materials and Methods) which represents the percentage of the signal power spectrum (mV^2^/Hz) of each oscillation in relation to the power spectrum of whole signal (mV^2^/Hz). The following graphs show the ratio of power that exists in the specific range for each oscillation [Delta (1–4 Hz), Theta (4–8 Hz), Alpha (8–12 Hz) and Beta (12–30 Hz) and Total Gamma (30–150 Hz)]. The rate of power was calculated in three conditions, control aCSF, high K^+^ aCSF and high K^+^ aCSF plus 2 μM diazepam, for each genotype. **(A)** Bar graph showing the rate of power of each frequency domain in heterozygous (left) and Rac1 cKO (right) brain slices in control aCSF, revealing that the most dominant oscillatory activity was the gamma (30–150 Hz) frequency domain in the heterozygous and Rac1 cKO acute brain slices across all conditions. [*n* = 5 slices from five Rac1 cKO mice; *n* = 5 slices from five heterozygous mice, heterozygous: one-way ANOVA, *F*_(1,10)_ = 3.067, *p* = 0.01 and Rac1 cKO: ordinary one-way ANOVA, *F*_(1,10)_ = 18.06, *p* = 0.01, comparison between groups and within groups with Tukey *post hoc* test]. **(B)** Bar graph showing the rate of power in delta (1–4 Hz) frequency domain. A trend for increase in high K^+^ aCSF and in K^+^ aCSF plus diazepam was observed in heterozygous brain slices. In Rac1 cKO brain slices, the rate of delta power was lower compared to heterozygous brain slices and unaltered among all three conditions [*n* = 5 slices from five Rac1 cKO mice; *n* = 5 slices from five heterozygous mice, one-way ANOVA, *F*_(1,15)_ = 1.239, *p* = 0.01, comparison between groups and within groups with Tukey *post hoc* test]. **(C)** Bar graph showing the rate of power in theta (4–7 Hz) frequency domain. A trend for increase in high K^+^ aCSF and in K^+^ aCSF plus diazepam was observed in heterozygous brain slices. In Rac1 cKO brain slices, the rate of theta power was lower compared to heterozygous brain slices and unaltered among all three conditions [*n* = 5 slices from five Rac1 cKO mice; *n* = 5 slices from five heterozygous mice, ordinary one-way ANOVA, *F*_(1,15)_ = 0.7201, *p* = 0.01, comparison between groups and within groups with Tukey *post hoc* test]. **(D)** Bar graph showing the rate of power in alpha (8–12 Hz) frequency domain. A trend for increase in high K^+^ aCSF and in K^+^ aCSF plus diazepam in heterozygous brain slices. In Rac1 cKO brain slices, the rate of theta power was lower compared to heterozygous brain slices and unaltered among all three conditions [*n* = 5 slices from five Rac1 cKO mice; *n* = 5 slices from five heterozygous mice, ordinary one-way ANOVA, *F*_(1,15)_ = 0.6602, *p* = 0.01, comparison between groups and within groups with Tukey *post hoc* test]. **(E)** Bar graph showing the rate of power in beta (13–30 Hz) frequency domain was lower in heterozygous than Rac1 cKO brain slices. In either genotype, the rate of power did not change in any of the three conditions [*n* = 5 slices from five Rac1 cKO mice; *n* = 5 slices from five heterozygous mice, ordinary one-way ANOVA, *F*_(1,15)_ = 3.778, *p* = 0.01, comparison between groups and within groups with Tukey *post hoc* test]. **(F)** Bar graph showing the rate of power in total gamma (30–150 Hz) frequency domain. A trend for increase was observed in high K^+^ aCSF plus diazepam in heterozygous brain slices, compared to high K^+^ or control aCSF. In Rac1 cKO brain slices the rate, of gamma power was lower compared to heterozygous brain slices and was unaltered in all three [*n* = 5 slices from five Rac1 cKO mice; *n* = 5 slices from five heterozygous mice, ordinary one-way ANOVA, *F*_(1,15)_ = 0.0478, *p* = 0.01, comparison between groups and within groups with Tukey *post hoc* test].

**FIGURE 8:**
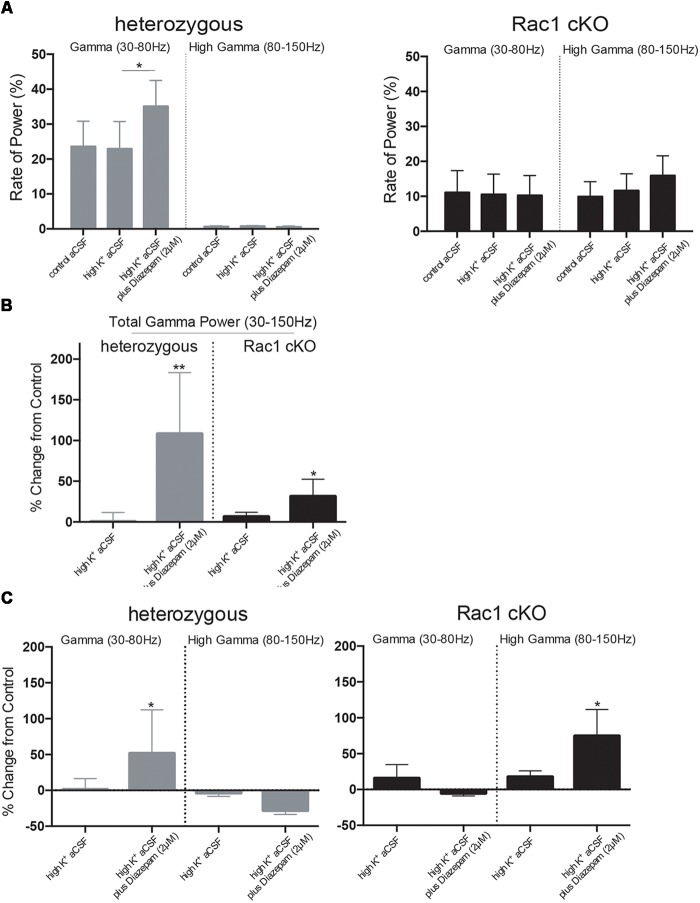
Disorganized gamma oscillations in Rac1 cKO brain slices. **(A)** Bar graph showing the rate of power in gamma (30–80 Hz) and high gamma (80–150 Hz), in heterozygous (left) and Rac1 cKO (right) brain slices. In heterozygous brain slices the rate of power gamma increased in high K^+^ aCSF plus diazepam [auto isxyei statistika?]. The rate of power of high gamma was significantly lower compared to rate of gamma. In Rac1 cKO brain slices, the rate of power gamma was unaltered among the three conditions and was decreased when compared to heterozygous brain slices. The rate of power of high gamma was significantly increased in Rac1 cKO compared to heterozygous brain slices (*n* = 5 slices from five Rac1 cKO mice; *n* = 5 slices from five heterozygous mice) [one-way ANOVA, *F*_(1,5)_ = 1.52, *p* = 0.01]. **(B)** Bar graph showing the overall effect of high K^+^ aCSF and high K^+^ aCSF plus diazepam on elicited total gamma oscillation (30–150 Hz) (% changes each condition from control aCSF). In high K^+^ aCSF plus diazepam, both heterozygous and Rac1 cKO brain slices show a significant increase on overall gamma power (*n* = 5 slices from five Rac1 cKO mice; *n* = 5 slices from five heterozygous mice) (two-tailed *t*-test, *p* < 0.01). **(C)** Bar graph showing the overall effect of high K^+^ aCSF and high K^+^ aCSF plus diazepam on elicited gamma power (30–80 Hz) and high gamma (80–150 Hz). In heterozygous brain slices, the power of gamma range (30–80 Hz), but not the high gamma, is enhanced, while in the Rac1 cKO brain slices the power of high gamma range (80–815 Hz) is enhanced (*n* = 5 slices from five Rac1 cKO mice; *n* = 5 slices from five heterozygous mice) (two-tailed *t*-test, *p* < 0.01), but not the power of gamma.

We next quantified the oscillatory activity in the range of delta (1–4 Hz), theta (4–7 Hz), alpha (8–12 Hz), beta (13–30 Hz), total gamma (30–150 Hz), gamma (30–80 Hz) and high gamma (80–150 Hz) band activity, using the rate of power metric. The rate of power (%) of each frequency domain revealed that the most dominant oscillatory activity was the gamma (30–150 Hz) frequency domain in the heterozygous and Rac1 cKO brain slices across all three conditions (Figures 7A,F and Table 1). The rate of total gamma power does not change in high K^+^ aCSF but it increases in the presence of diazepam in both genotypes (Figures 7F, 8B and Table 1). This is in accordance with previous reports linking the function of GABA-A receptors (which are positively modulated by diazepam) to total gamma power (Whittington et al., 1995; Traub et al., 1996).

**Table 1:** Rate of power (%).

	Heterozygous				Rac1 cKO			
		
	Control aCSF	High K^+^ aCSF	High K^+^ aCSF plus Diazepam (2 μM)	*n*	Control aCSF	High K^+^ aCSF	High K^+^aCSF plus Diazepam (2 μM)	*n*
Delta (1–4 Hz)	0.2396 ± 0.2371	0.02731 ± 0.01909	0.04469 ± 0.03017	*Five slices from five animals*	0.0003338 ± 0.0001266	0.0003598 ± 8.614e-005	0.0004203 ± 5.636e-005	*Four slices from four animals*
Theta (4–7 Hz)	0.1153 ± 0.1135	0.02288 ± 0.01787	0.03813 ± 0.02539	*Five slices from five animals*	0.0003597 ± 0.0001315	0.0003837 ± 8.217e-005	0.0004471 ± 4.147e-005	*Four slices from four animals*
Alpha (8–13 Hz)	0.002457 ± 0.001435	0.03046 ± 0.02486	0.04814 ± 0.03201	*Five slices from five animals*	0.0005712 ± 0.0001731	0.0006159 ± 0.0001268	0.0006824 ± 3.717e-005	*Four slices from four animals*
Beta (13–30 Hz)	0.1555 ± 0.09180	0.09185 ± 0.05178	0.2041 ± 0.1109	*Five slices from five animals*	0.8216 ± 0.5611	0.8320 ± 0.5164	0.7018 ± 0.4257	*Four slices from four animals*
Gamma (30–80 Hz)	23.55 ± 7.268	22.92 ± 7.796	35.04 ± 7.431	*Five slices from five animals*	11.11 ± 6.251	10.56 ± 5.793	10.29 ± 5.658	*Four slices from four animals*
High Gamma (80–150 Hz)	0.6732 ± 0.2058	0.8279 ± 0.1637	0.8279 ± 0.1637	*Five slices from five animals*	9.955 ± 4.246	11.65 ± 4.798	15.93 ± 5.667	*Four slices from four animals*
Total Gamma (30–150 Hz)	24.05 ± 7.108	23.55 ± 7.666	35.44 ± 7.250	*Five slices from five animals*	20.83 ± 7.958	21.95 ± 8.021	25.86 ± 8.379	*Four slices from four animals*


In the heterozygous brain slices, the rate of delta, theta and alpha power showed a trend toward increase in high K^+^ aCSF (Figures 7B–D and Table 1), while the rate of beta power was unaltered, compared to control aCSF (Figure 7E and Table 1). In the presence of diazepam in high K^+^ aCSF, the rate of delta and theta power was increased (Figures 7B,C and Table 1), similar to the rate of gamma power (Figure 7F and Table 1), while the rate of beta power was again unaltered (Figure 7E and Table 1).

In the Rac1 cKO brain slices, the rate of delta, theta and alpha power was significantly reduced over the three conditions compared to heterozygous brain slices (Figures 7B–D and Table 1). On the other hand, an enhanced peak rate of power beta appeared in the Rac1 cKO brain slices, which did not change in high K^+^ aCSF or in the presence of diazepam and remained slightly increased compared to heterozygous brain slices (Figure 7E and Table 1). Furthermore, the Rac1 cKO brain slices displayed lower peak rate of total gamma band power compared to heterozygous, in control aCSF (Figures 7A,F and Table 1). However, in Rac1 cKO brain slices, the peak of rate of gamma band did not change in high K^+^ aCSF or in the presence of diazepam (Figure 7F and Table 1). Together, these findings indicate there was a lack of capacity of Rac1 cKO cortical neuronal networks to synchronize at lower frequencies and to modulate their oscillatory domains in the presence of increased excitability and diazepam.

### Gamma Oscillations Are Disorganized in the Rac1 cKO Brain Slices

Complexity and/or reduction in the amplitude of gamma responses has been revealed by many clinical studies of SCZ (Spencer et al., 2003, 2004; Uhlhaas and Singer, 2010). Our spectral analysis revealed that a single predominant peak occurs between 40 and 60 Hz in heterozygous brain slices (Figure 6A) which was increased in high K^+^ aCSF plus diazepam. On the contrary, in Rac1 cKO brain slices, the power spectral density showed peaks in multiple frequency ranges, one at 40–60 Hz and a second one at 100 Hz (Figure 6B). Both peaks were not modulated by high K^+^ aCSF and the further addition of diazepam (Figure 6B). High frequency of gamma band is known to span from roughly gamma (30–80 Hz) to high gamma (>80 Hz) (Moran and Hong, 2011). We computed the rate of power gamma (30–80 Hz) and high gamma (80–150 Hz) separately and found significantly decreased rate of power gamma in Rac1 cKO, compared to heterozygous brain slices (Figure 8A), in all three conditions.

Furthermore, bath perfusion with diazepam in high K^+^ aCSF significantly increased the amplitude and the total gamma power in heterozygous (fold change from control aCSF: 108.2 ± 75.33%) and Rac1 cKO (fold change from control aCSF: 31.36 ± 21.21%) genotypes (Figure 8B). Specifically, this increase in heterozygous slices is derived from the increased gamma band (30–80 Hz) in high K^+^ aCSF plus diazepam when compared to control aCSF (Figure 8C). In Rac1 cKO slices, the major part of the increase in high K^+^ aCSF plus diazepam condition is derived from the increase of the high gamma band (80–150 Hz) (Figure 8C). Consequently, the developmental decrease of interneuron numbers in Rac1 cKO cortical neuronal networks are characterized by altered gamma power as well as reduced low frequency oscillations.

## Discussion

In this study, we examined the effect of decreased inhibition throughout postnatal life on the functional organization of adult local cortical circuits, in brain slices. We show that this decrease results in increased synaptic transmission, decreased LTP and enhanced spontaneous activity. Furthermore, spontaneous oscillatory activity displays significant abnormalities in Rac1 cKO mice, particularly a significant decrease of the range of low frequencies, alteration of the gamma frequency range (30–80 Hz) and an aberrant peak at the high gamma frequency range (80–150 Hz).

### Physiological Changes in Rac1 cKO Mice Cortex

As a consequence of the specific ablation of Rac1 from Nkx2.1-expressing MGE-derived cells, we observed a severely reduced number of all MGE-derived neurons in the adult brain (Vidaki et al., 2012). In addition, we have established that the significant decrease in cortical interneurons causes adaptations in several features of the mature glutamatergic transmission in the adult prefrontal cortex (PFC), such as the PPR at 20 Hz, LTP induction, dendritic spines and NMDA receptor subunit expression. Furthermore, we found that increasing GABA receptor function during early postnatal life reverses the defect in dendritic morphology (Konstantoudaki et al., 2016).

Our data demonstrate that in the presence of reduced inhibitory activity postnatally, the synaptic plasticity and stimulus-induced recurrent activity are affected (Figures 2, 3). Reports in the literature using *in vitro* slice models of epilepsy, including electrical kindling in slices, high K^+^, zero Mg^2+^, zero Ca^2+^, 4-aminopyridine-induced and bicuculline-induced seizures, have revealed this increased burst activity and spontaneous events (Gutiérrez et al., 1999; Hochman, 2012). Our study shows that in high K^+^ and zero Mg^2+^ environments, a pronounced susceptibility and rapid saturation in the emergence of spontaneous activity is observed in the Rac1 cKO (Figures 4, 5).

### Deficits in Neuronal Oscillatory Activity in Rac1 cKO Mice

Even small changes in the balance between excitation and inhibition can result in runaway excitability (Chagnac-Amitai and Connors, 1989), disruption of sensory responses (Nelson, 1991) and alteration of experience-dependent plasticity (Hensch, 2005). Spontaneous activity oscillations are also critical for the maturation and plasticity of cortical networks at several developmental stages (Katz and Shatz, 1996; Ben-Ari, 2001; Khazipov and Luhmann, 2006; Luhmann et al., 2016). Neuronal oscillation is rhythmic activity within a narrow frequency range and represents an essential mechanism for enabling coordinating activity during normal brain function.

Oscillations in different frequency ranges have been associated with various cognitive functions and underlying neurobiological mechanisms. Electroencephalography (EEG) records cortical oscillations and they are typically described as low frequency bands at delta (1–4 Hz), theta (4–8 Hz), alpha (8–12 Hz), and beta (12–30 Hz) and high frequency oscillations at gamma (30–80 Hz) and high gamma bands (>80 Hz) (Moran and Hong, 2011; Sun et al., 2011). Although the majority of the studies analysing oscillations were done in either awake or anesthetized animals, some studies have been performed *in vitro*. These studies in acute brain slices have revealed that oscillatory population activity could be generated by isolated local circuits. Therefore, these studies justify the use of brain slices as an experimental approach for investigation of oscillations that resemble the *in vivo* rhythmic activity (Whittington et al., 1995, 1997; Cunningham et al., 2003; McNally, 2013; Rebollo et al., 2018).

Oscillations in the delta range (1–4 Hz) is limited and may relate to coding of sensory stimuli (Lakatos et al., 2005, 2008). Theta oscillations (4–7 Hz) are evident in the hippocampus but occur also in the ento- and the perirhinal, the prefrontal, somatosensory and visual cortex, and superior colliculus (Raghavachari et al., 2006; Tsujimoto et al., 2006). Theta activity has been implicated in the memory process and is particularly effective in inducing LTP (Pavlides et al., 1988; Huerta and Lisman, 1993; Hölscher et al., 1997). Alpha oscillations (8–12 Hz) are very prominent in the thalamus but have also been recorded in all cortical and subcortical areas (Steriade et al., 1993; Başar et al., 1997). Beta frequency oscillations (13–30 Hz) are related to the perception of environmental novelty in rodents (Berke et al., 2008) and their generation has been associated with particular neurotransmitter systems, including NMDA and GABA-A receptor activities (Traub et al., 2004; Yamawaki et al., 2008). Oscillations in the gamma range have been recorded in several cortical areas and have been correlated with cognitive functions (Tiitinen et al., 1993; Uhlhaas et al., 2008). GABAergic interneurons play the role of pacemakers in the generation of high frequency oscillations by producing rhythmic inhibitory post synaptic potentials in pyramidal neurons (Cobb et al., 1995; Wang and Buzsáki, 1996; Konstantoudaki et al., 2014). Particularly, PV+ interneurons are correlated with fast-spiking cells and their activity is essential for generation and synchronization of gamma rhythms in mice, *in vivo* (Sohal et al., 2009). Alterations in fast and slow oscillations have been associated with FS interneurons (Joho et al., 1999).

In our mouse model, we observed decreased power in the slow oscillations, including delta, theta and alpha oscillations, thus linking these oscillations with normal inhibitory function. In another transgenic line with interneuron deficits (Batista-Brito et al., 2009), dysregulation in the delta and theta oscillations is correlated with interneuron maturation. In addition, we have shown that the developmental decrease of interneurons correlates with impaired synaptic plasticity and NMDA subunit levels in the adult cortex (from Konstantoudaki et al., 2016) and increased beta power (this report), making a more direct link between the reduction in MGE-derived interneurons and concurrent increases in beta power. Finally, the Rac1 cKO exhibits a decreased number of PV+ and SST+ interneurons by 50% (Vidaki et al., 2012) and reduced gamma oscillations *in vitro* (data from this work Figures 7, 8), further corroborating the fundamental role of these subpopulations of interneurons in the generation of synchronized gamma rhythms.

### Synaptic Transmission and Neuronal Oscillations

Synaptic GABA-A receptor-mediated inhibition may be sufficient to generate network oscillations *in vivo* (Whittington et al., 1995; Traub et al., 1996). In the presence of high extracellular K^+^ concentration, multiple other mechanisms (ionic transporters Na^+^- K^+^ -2Cl^-^ (NKCC1) and K^+^ -Cl^-^ (KCC2), extra-synaptic GABA(A) receptors, and the GAT-1 transporter) could lead to the development of gamma bursts *in vitro* (Subramanian et al., 2018). Our data shows that heterozygous slices at high K^+^ aCSF show increased amplitude of gamma power in the presence of diazepam. However, GABA-A receptor activation does not seem to be efficient in order to increase gamma power in a cortex with significantly fewer interneurons, as is the case of Rac1 cKO, suggesting a disruption of gamma oscillatory activity.

With regards to the glutamatergic system, inhibition of NMDA receptor function induces aberrant high frequency oscillations throughout cortical and subcortical networks (Pinault, 2008; Rebollo et al., 2018). Our Rac1 cKO mice display impaired synaptic transmission and NMDA-dependent LTP, reduced gamma frequency range (30–80 Hz) and induced aberrant high gamma frequency oscillations (80–150 Hz) in the cortex. These results agree with the findings that inhibition and excitation are in close connection and link studies on neuronal oscillations and the mechanisms that influence E/I balance. Finally, reduction of theta and gamma activity have been correlated with decreased LTP (Huerta and Lisman, 1993; Wespatat et al., 2004; Stephan et al., 2009; Kalweit et al., 2017). This view is consistent with our results since LTP is reduced along with the beta and gamma powers of oscillatory activity.

### Mice With Decreased Interneuron Function and Alterations in Oscillatory Activity

Several animal models exist in which modulation of specific molecules in interneurons alters their functional attributes and the mature neuronal networks. Early removal of Nkx2.1 or Lhx6 results in decreased numbers of PV and SST-expressing interneurons and the emergence of prolonged abnormal bursting activity in the cortex, as measured by EEG recordings (Butt et al., 2008; Neves et al., 2012). A number of transgenic mice exist with decreased numbers of various subpopulations of cortical interneurons with resulting alterations in oscillatory activity (Batista-Brito et al., 2009).

Here, we show that the Rac1 cKO transgenic mouse line that exhibits a 50% decrease of PV and SST interneurons (Vidaki et al., 2012) demonstrate reduced low frequency oscillations, increased beta power and aberrant gamma oscillations. Therefore, our study reinforces the connection between PV and SST interneurons with proper gamma frequency oscillations, while also providing a link between these two interneuron subtypes with the low frequency and beta oscillations. Future experiments could further investigate the functional mechanisms via which this developmental decrease of interneurons affects the oscillatory activities of local neuronal networks.

### Disrupted E/I Balance and Oscillations in Neuropsychiatric Disorders

Alterations in E/I balance can be caused by changes in interneuron numbers (Benes, 1991; Selby et al., 2007; Sakai et al., 2008; Peñagarikano et al., 2011; Fung et al., 2014) or reduced expression of markers of interneuron populations without changes in cell numbers *per se* (Hashimoto et al., 2003; Fatemi et al., 2008a,b; Volk and Lewis, 2013; Donato et al., 2014; Hanno-Iijima et al., 2015; Enwright et al., 2016; Filice et al., 2016). Shifts in the E/I balance as well as modifications in neuronal oscillations, especially the well-studied gamma frequency band, are observed in many psychiatric disorders, including ASD, SCZ, bipolar disorder and depression (Rubenstein and Merzenich, 2003; Klempan et al., 2007; Sakai et al., 2008; Luscher et al., 2010; Marin, 2012; Fung et al., 2014; Gao and Penzes, 2015; Canitano and Pallagrosi, 2017; Selten et al., 2018). It has been hypothesized that aberrant gamma oscillations underlie deficits in higher order cognitive processes (Tiitinen et al., 1993; Spencer et al., 2003, 2004; Cho et al., 2006; Uhlhaas et al., 2008; Uhlhaas and Singer, 2010, 2012).

## Conclusion

Our study contributes toward the elucidation of synaptic physiology of local cortical circuits. We demonstrate that the capacity of local neuronal synchronization is reduced in the cortex when cortical interneurons are significantly decreased. It is tempting to speculate that altered synchronization over a larger cortical region follows decreased oscillatory frequencies within local circuits that in turn ensue from the reduction in interneuron numbers throughout postnatal life. Therefore, our data may enhance to understand the interneuron dysfunction often observed in animal models and clinical studies.

## Author Contributions

KK, KS, and DK designed the experiments and wrote the paper. KK, XK, ST, and KS performed the experiments. KK performed the oscillation analysis. KK, DK, KS, XK, and ST edited the paper.

## Conflict of Interest Statement

The authors declare that the research was conducted in the absence of any commercial or financial relationships that could be construed as a potential conflict of interest.

## Footnotes

1 https://github.com/nbluoc/kk
